# Human pluripotent stem cell registry: Operations, role and current directions

**DOI:** 10.1111/cpr.13238

**Published:** 2022-05-06

**Authors:** Andreas Kurtz, Nancy Mah, Ying Chen, Antonie Fuhr, Sabine Kobold, Stefanie Seltmann, Sabine C. Müller

**Affiliations:** ^1^ Fraunhofer Institute for Biomedical Engineering Sulzbach Germany; ^2^ BIH Center for Regenerative Therapies Charite Universitätsmedizin Berlin Berlin Germany

## Abstract

The human plutiripotent stem cell registry (hPSCreg) is a global database for human embryonic and induced pluripotent stem cells (hESC, hiPSC). The publicly accessible Registry (https://hpscreg.eu) was set up to provide a transparent resource of quality‐assessed hPSC lines as well as to increase reproducibility of research and interoperability of data.

**Objectives:**

In this review, we describe the establishment of the Registry and its mission, its development into a knowledgebase for hPSC and the current status of hPSC‐focussed databases. The data categories available in hPSCreg are detailed. In addition, sharing and hurdles to data sharing on a global level are described.

**Conclusions:**

An outlook is provided on the establishment of digital representatives of donors using hybrids of data and hPSC‐based biological models, and how this can also be used to reposition databases as mediators between donors and researchers.

## Graphical abstract

hPSCreg as a data hub for pluripotent stem cells: Key utility and function.

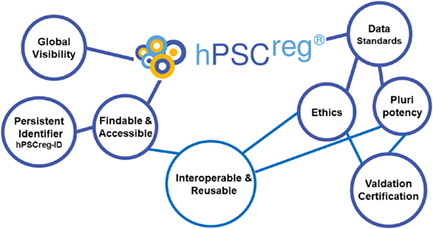

## 1 THE HUMAN PLURIPOTENT STEM CELL REGISTRY – A EUROPEAN INITIATIVE

The European Human Embryonic Stem Cell Registry (hESCreg) was founded in 2007 by the European Commission (EC) and generously funded by the EU's Sixth Framework Programme.^1^ Its original remit was to monitor and inform the EC about the state of human embryonic stem cell research carried out in the EU. Following the discovery of induced pluripotent stem cells (iPSC) in 2006 and their subsequent rapid adoption by the research community, this lead to the inclusion of both human ESC and iPSC in the Registry and prompted a name change to the ‘human pluripotent stem cell registry’ (hPSCreg), which was funded by the EC in the Framework 7 Programme and subsequently the HORIZON 2020 Programme. hPSCreg is poised for another round of EC funding in the Horizon Europe Programme, demonstrating the continued value of hPSCreg to the EC and the stem cell community at large as a key resource infrastructure for innovative, emergent technologies and novel therapies arising from hPSC applications. As hPSCreg aims at serving the research community, its integration and open cooperation with the stem cell community and diverse stakeholders is of utmost importance to translate the stem cell field's progress into useful data resources. HPSCreg has therefore established a Committee of National Representatives (CNR) to engage with national developments in the field. In addition, hPSCreg cooperates with international stakeholder initiatives and resources, including the International Stem Cell Banking Initiative (ISCBI; https://iscbi.org), the International Stem cell Alliance (ISCA; http://www.stem-cell-network.net/), the International Society for Stem Cell Research (ISSCR; https://www.isscr.org/) and the European Bank for iPSC (EBiSC; https://ebisc.org), the European Joint Programme – Rare Diseases (EJP‐RD; https://www.ejprarediseases.org/), the European Consortium for Communicating Gene and Cell Therapy Information (EuroGCT; www.eurogct.org).

## 2 HPSCREG – A PLURIPOTENT STEM CELL KNOWLEDGEBASE

hPSCreg is firmly established as a global registry for hESC and hiPSC lines.^2^, ^3^ Registering lines at hPSCreg yields primary benefits according to the FAIR data principles.^4^ These include promoting global visibility of hPSC lines and their findability across multiple resources (such as other databases or publications) by using a unique standard nomenclature to reference pluripotent stem cell lines, thereby reducing cell line misidentification.^5^ hPSCreg records key cell line data through the use of ontologies and community‐accepted standards, thereby creating cell line datasets with rich metadata annotations that render the data both human and machine accessible and increase its interoperability between disparate resources. The cell line data are freely accessible (https://hpscreg.eu) and can be used by stakeholders to compare and identify the cell lines of interest, for example, hPSC lines from a particular disease or project. Finally, hPSCreg promotes transparency in the ethical provenance of the hPSC lines and the biological properties to demonstrate pluripotency of the cell lines. Certificates issued by hPSCreg, attesting to the ethical provenance and pluripotency of hPSC lines, are recognized by the EC to fulfil ethical requirements for EC‐funded research. Currently, about 7400 hPSC lines have been registered at a persistently high rate (Table 1).

**TABLE 1:** Registration of new ESC and iPSC lines in hPSCreg (2017–Dec 2021)

Year	ESC	iPSC
2017	58	1192
2018	22	775
2019	52	1355
2020	102	1150
2021 (Jan–October)	55	911

Building on its position as an international registry for hPSC lines, hPSCreg established a database exclusively for clinical studies that use hPSCs or hPSC‐derived cells for interventional treatment.^6^ The database is actively maintained and provides an overview of the translational progress stem cells have made towards clinical application. To complete the translational path from research to clinical application, hPSCreg will further expand the registry to include data on ‘regulatory primed’ hPSC lines, that is, hPSC lines that are intended for clinical use. The regulatory primed hPSC registry will record additional critical information on the lines, especially as it pertains to the ethical provenance and biological safety of the cells destined for therapeutic purposes. Coupled with its unique clinical studies database exclusively for hPSC therapeutic products, the Registry is in an excellent and ideal position to host the full spectrum of hPSC data starting from an extensive dataset of hPSC lines for research applications and ending with clinical studies that involve hPSC lines or hPSC‐derived cell products.^6^

## 3 DATA CATEGORIES IN HPSCREG

Information provided by hPSCreg include data on (i) pluripotent stem cell lines, (ii) clinical studies and other research performed using human PSC lines, and (iii) on regulatory, legal and ethical frameworks for pluripotent stem cell research (Table 2). Importantly, a unique persistent identifier is assigned to each hPSC lines upon registration in hPSCreg, which is generated automatically based on a standard nomenclature.^5^ The generators of the cell lines register the cell lines individually, or larger resources such as hPSC‐banks may register cell lines and data using a specifically adapted application programming interface (API). These are annotated, enriched and linked by hPSCreg, for example with publications, projects or clinical trial information. As much as possible, hPSCreg uses standards such as taxonomies, nomenclatures and ontologies for registered data to improve interoperability.

**TABLE 2:** Data categories in hPSCreg

hPSC lines	Clinical studies and projects	Legal frameworks
Ethical provenance	Study topic	National regulations
Establishment and cultivation	Study phase and site	EU ethical & legal framework
Cell line characteristics	Cell lines used	Data protection regulations

## 4 HUMAN PSC‐RELATED DATA

**FIGURE 1:**
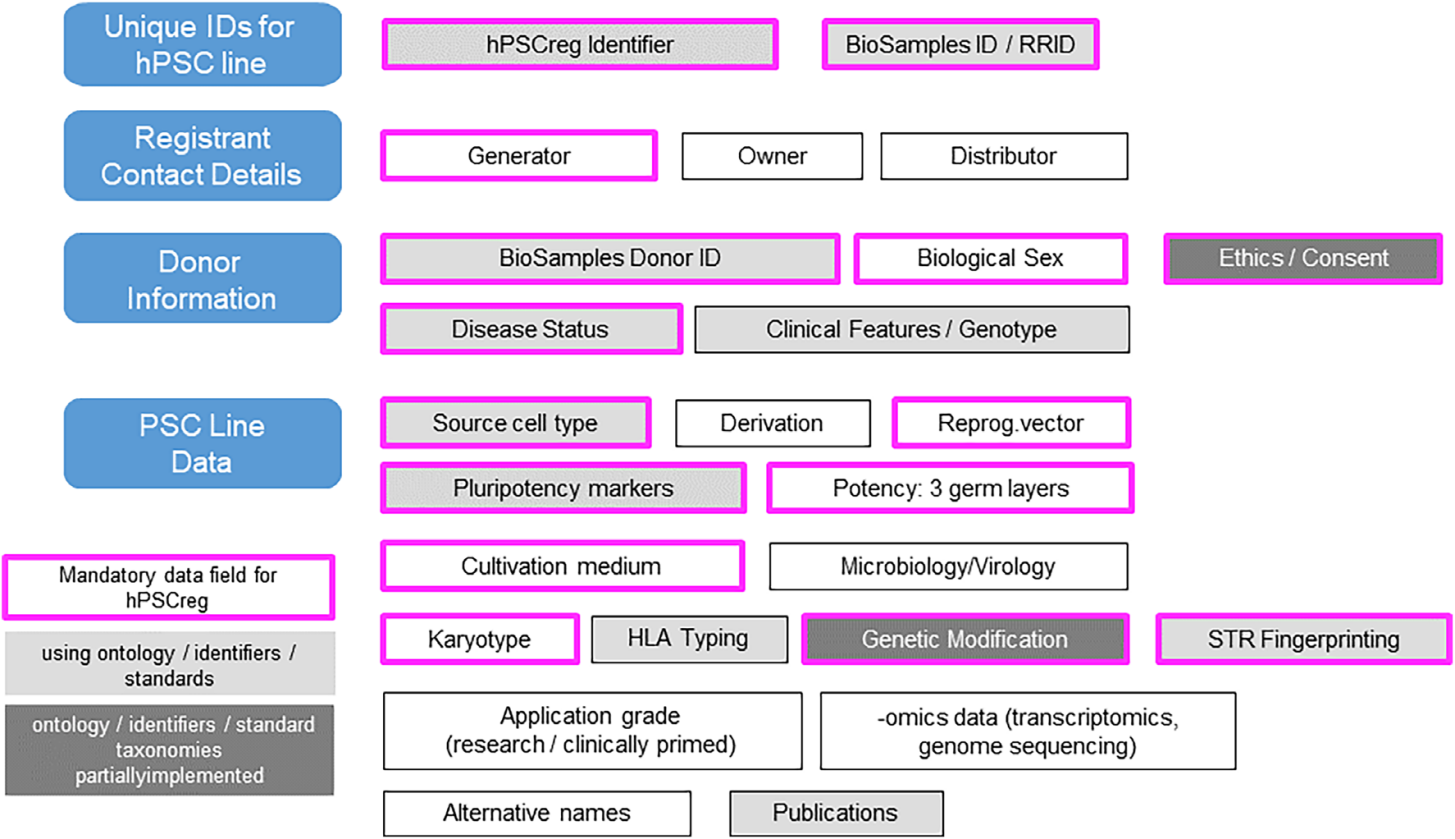
Data categories for hPSC lines for cell line registration. Data mandatory for assessment and validation are in the coloured boxes. Dark grey boxes indicate lack or high diversity of available ontologies, taxonomies or data standards

To register a cell line in hPSCreg it is required to include data on ethical provenance of the cells, and data on their authenticity, biological characteristics and scientific quality (Figure 1). Some of the data are mandatory to describe the characteristics and biological properties of the cells. These data categories are based on international standards for cell characterization.^7^, ^8^ In addition, information on the ethical provenance of the cells is required.^9^, ^10^, ^11^ These mandatory data are further annotated using diverse data sources including publications, projects and feedback from users. For example, if original hPSC lines are used to generate genetically modified lines (termed subclones in hPSCreg), these will be linked in the Registry and progeny identified by the standard nomenclature and its derived identifier.^5^

Mandatory data are in general needed to determine the ethical provenance of the cells, the derivation and cultivation conditions, authenticity, clinical features of the donor, the pluripotency status, and genetic constitution and genetic modifications. Data are assessed before data are formally validated by hPSCreg, thereby providing a measure of quality based on completeness and relevance of provided data. This includes a check of ethical provenance, namely, proof that informed consent procedures and adequate ethics approval processes were followed. Ethial provenance is manually assessed as these differ nationally, change over time and are distinct between hESC and hiPSC lines. Certification of data completeness and quality is done upon request on a formalized and partially automated process of the data required for certification. For example, hPSCreg ‐certification of hPSC lines is necessary for its use in research funded by the European Commission, which requires the application of the ethical standards relevant for the active framework programmes. Currently, 370 hPSC lines registered in hPSCeg have been certified. The procedures guiding data assessment and certification are available at the hPSCreg portal (https://hpscreg.eu/about/documents-and-governance).

Data deposited in hPSCreg are further annotated and linked to enrich the available information content. This includes the usage of lines in published research, in a funded project or a clinical study. Links to hPSC‐resources such as EBiSC, to the European Bioinformatics Institute (EBI) via the unique Biosamples ID (https://www.ebi.ac.uk/biosamples/), and to the Cellosaurus database^12^ provide further expansion into relevant data ecosystems covering genetic data, donor data or applications.

Access to genetic data such as whole‐genome sequences (WGS), single‐nucleotide polymorphism profiles (SNPchip) or transcriptome data are of highest interest for assessment of hPSC lines, including for assessing their risks for clinical use, or for personalized medicine. Most of these data are stored in large repositories such as the European genome phenome archive (https://ega-archive.org/) or GenBank and NCBI (https://www.ncbi.nlm.nih.gov/genbank/). Data are also stored locally in dedicated resources. Since genetic data, including WGS, STR or SNPchip data constitute sensitive personal data, access and sharing must be controlled. Thus, facilitating access for research and protecting privacy are challenging tasks, which makes open search, filter and comparison difficult. Solutions towards facilitiating genetic datause while at the same time protecting privacy include genome beacons, developed by the Global Alliance for Genomics and Health (*GA4GH*) (https://beacon-project.io/). hPSCreg has established a data access committee (DAC) to enable access to genetic data associated with registered hPSC lines

## 5 CLINICAL STUDIES DATABASE

The hPSCreg clinical study database is unique as it is selectively focussing on interventional studies using products based on hPSC. Clinical study data are collected on a regular basis from a multitude of publicly accessible sources including clinical trial registries worldwide [e.g. clinicaltrials.gov, from the US National Library of Medicine and the International Clinical Trials Registry Platform (ICTRP); https://trialsearch.who.int/ of the World Health Organization (WHO)]. In addition, public information in the press is obtained by continuously examining Google News Alerts and press databases containing the terms ‘stem cell’ or ‘stem cells’ and ‘clinical trials’. By screening the sponsor's web pages, additional information on the respective clinical trials is evaluated. Furthermore, topic specific newsletters are screened and scientific literature searches are carried out.

Following a general search for clinical studies involving hPSCs, basic study data are extracted from the WHO Registry (https://trialsearch.who.int/). Basic study data originate from the Trial Record Data Set (TRDS) fields outlined by the WHO, whereas value‐added data constitute specific data or metadata manually added to the clinical trial record. In the case of clinical studies involving hPSCs, information pertinent to the respective cell products, such as the cell line provenance from the source hPSC line to its hPSC‐derived therapeutic product, is not a mandatory information in the TRDS. This value‐added information is manually collected by hPSCreg staff through public sources. Currently, the hPSC source line is not mandatory because this information is not available for some hiPSC‐based cell products; however, hPSCreg aims at making this source cell information mandatory for new trials, and retrospectively for already registered trials. All these data are saved in the hPSCreg platform (https://hpscreg.eu/browse/trials). Although the database makes comprehensive data accessible, other interesting information is not available. For example, more detailed information on the underlying studies could be provided including a description of the therapeutic approach and technical principles of the envisaged therapies. Because such details are mostly not publicly available, they must be provided by persons involved in the clinical studies. To facilitate this, direct interaction with the study PIs also facilitating and additional validation step needs to be implemented. Finally, Qualified Investigators of clinical studies are contacted to check the veracity of the data, resulting in an up‐to‐date record of the clinical studies. However, the source hPSC‐line and information on the therapeutic cells remain often not available and are not disclosed. Study investigators are encouraged to disclose this information, which would greatly improve transparency and comparability of study results.

## 6 CURRENT DATA AND DATA ACCESS

As of Feburary 2022, there were 96 clinical trials using cells derived from hPSCs, including long‐term observational studies with patients from previous trial registered on the hPSCreg database. Forty eight studies are using material derived from hESC. Forty‐five clinical trials are conducted using cells based on hiPSC. Two studies apply cells derived from human parthenogenetically produced pluripotent stem cells (hpPSC), and one study uses cells based on nuclear transfer (SCNT) (NT‐hESC) (Table 3). The studies are being carried out in 13 different countries.

**TABLE 3:** A total of 11 different types of clinical indication grouped by different ICD‐10 chapters and associated disease areas, including four clinical trials for COVID‐19 treatment

Disease category	hESC	hiPSC	hpSC	SCNT	Summary
Arthropathies *Osteoarthritis*		1			1
Diseases of the blood and blood‐forming organs and certain disorders involving the immune mechanism *Acute myeloid leukaemia* *Beta thalassemia* *Aplastic anaemia* *Thrombocytopenia*		4			4
Diseases of the circulatory system *Ischaemic heart disease* *Ischaemic cerebrovascular accident* *Cardiomyopathy* *Congestive heart failure*	3	10			13
Diseases of the eye and adnexa *Dry age‐related macular degeneration (AMD)* *Wet AMD* *Stargardt disease* *Myopia* *Retinitis pigmentosa* *Limbal stem cell deficiency* *Dystrophies primarily involving the retinal Pigment epithelium* *Bullous keratopathy*	24	7		1	32
Diseases of the genitourinary system *Adhesions of uterus* *Interstitial cystitis*	2				2
Diseases of the musculoskeletal system and connective tissue *Meniscus derangement*	1				1
Diseases of the nervous system *Parkinson's disease* *Motor neuron disease* *Multiple sclerosis* *Epilepsy*	4	3	2		9
Endocrine, nutritional and metabolic diseases *Diabetes Type 1* *Primary ovarian failure*	8	1			9
Injury, poisoning and certain other consequences of external causes *Spinal cord injury* *Bone marrow graft rejection* *Meniscus derangement* *Tear of articular cartilage*	3	3			6
Neoplasms *Lung cancer* *Malignant neoplasms (general)* *Acute myeloid leukaemia* *Head and neck cancer* *chronic lymphocytic leukaemia* *Breast cancer* *Ovarian cancer* *non‐Hodgkin lymphoma*	1	14			15
Provisional assignment of new diseases of uncertain aetiology or emergency use *Covid 19 (lung injury)*	2	2			4
Total	48	45	2	1	96

*Note*: More details are available at https://hpscreg.eu/browse/trials.

Although data are open and accessible, improved search and filter and analytic capabilities (e.g. by status, indication, therapeutic cell type, etc.) still need to be implemented. Similarly, filtering and statistical functions are very useful for the cell line database for predefined standard attributes like regulatory primed PSC lines, commercial use, starting cell type or reprogramming method. These are currently being implemented. For that, user feedback from questionnaires was obtained, which identified cell lines‐specific attributes such as genetic constitution and variation, availability of clinical donor data as key search items. Moreover, hPSCreg is aiming to implement a more elaborate data scheme to accommodate the broad and more detailed information required to assess and compare hPSC lines intended for clinical application. These regulatory primed lines require a more elaborate control of their generation, manufacturing, quality and function than research grade lines, which additionally divert between different regulatory environments.

## 7 INFORMING ON NATIONAL LEGAL AND REGULATORY FRAMEWORKS IN HPSCREG

The national regulations for hESC and hiPSC research and application vary. In addition, some international agreements and funding mechanisms are imposed, for example the European Commission issued specific guidelines for research funding for projects involving hESC,^13^ while at the same time following the subsidiary principle regarding national law. Similarly, federal funding in the USA is limited to hESC lines listed in the NIH human embryonic stem cell registry (https://grants.nih.gov/stem_cells/registry/current.htm). The national legal and regulatory frameworks of countries from which hPSC lines are registered in hPSCreg are available on the hPSCreg portal (https://hpscreg.eu/map). This information is regularly completed and updated.

## 8 ISSUES AFFECTING DATA SHARING

One of hPSCreg's key goals is to make data findable, accessible, interoperable and reusable (FAIR) (https://www.go-fair.org/fair-principles/). A main component for FAIRification is the use of a persistent unique identifier (ID). This ID allows users to find cell lines and data, and to share their own data with that of others using the same cell line. Usage of a unique, centrally registered identifier reduces risks for reproducibility in research makes data sustainable and findable. Other factors affecting data sharing are the application of common standards for the characterization of donor and cell features, but also for data types and formats. For example, the multiple genetic and clinical data formats require the consistent usage of a wide spectrum of standards (Table 4). In addition, non‐standard information, sequence strings, free text or assay results needs to be accommodated but may not be useful in terms of machine readability or data comparison. The data standard issue for stem cells has been taken up by the International Standards Organization (ISO) as a critical topic.

**TABLE 4:** Genetic and clinical data – formats and standards

Genetic data	
Type	Example
Gene	HGNC nomenclature
Karyotype	International System for Human Cytogenetic Nomenclature (ISCN)
Single‐nucleotide polymorphism (SNP)	dbSNP reference SNP number (rs#)
Short‐tandem repeat (STR), HLA‐type, SNP pattern	STRAND working group nomenclature, forensic, STRBase, massive parallel sequencing (MPS)‐related formats, HLA‐nomenclature
DNA sequencing	Nucleotide sequence (IUPAC nucleic acid notation); raw sequence formats (FASTA, FASTQ); aligned sequence formats (BAM, CRAM)
Phenotype/Clinical feature (donor)	Disease ontology (DOID), online mendelian inheritance in me\an (OMIM), Human Phenotype Ontology (HPO), international classification of diseases (ICD), SNOMED, Orphanet Rare Disease Ontology (ORDO), HGVS nomenclature
Phenotype/Clinical feature (cell line)	HGVS nomenclature, Model function

Thirdly, sharing requires a permissible legal framework and pathway to allow international use of data for research. This is especially important when sensitive personal data are involved and potential privacy issues affected. For example, sharing of genetic data requires specific access and management procedures. Genetic data can be used to re‐identify the donor of the cell line and thus are often considered sensitive personal data. This includes also authentication data such as high resolution STR, HLA types or DNA sequencing. While access for research is usually possible, also under the General Data Protection Directive (GDPR) of the European Union, unintended use of the data must be prevented. While the EU permits export of genetic data in principle, especially into countries with equivalent data protection frameworks (Regulation [EU]2016/679), other countries do not allow data export and thus sharing of raw data.

## 9 STEPS TAKEN TO FACILITATE STEM CELL DATA SHARING

A globally integrated information resource would be of high benefit and interest for the community. The hPSCreg aims to cover as many hPSC lines as possible and reaches out, links and exchanges data with other platforms and cell banks globally. Some of the hurdles for integration of different data resources have been outlines above. These include the use of a persistent unique identifier, which is prerequisite for data sharing and unambiguous identification of lines. Other hurdles include variable data platforms, the use of different data standards and taxonomies, regulatory and legal restrictions in access and data sharing. With the growing amount of PSC lines generated by different laboratories all over the world (Table 5), stem cell banks and registries are expected to provide necessary data on individual stem cell lines. However, the exchange of data among institutions is not a trivial matter, since the scientific reproducibility of the stem cells can vary, particularly in hiPSCs generated by different methods. Depending on available information is problematic for both basic studies and clinical application.^14^ Many efforts have been made to make the data flow more efficient and accessible among different stem cell repositories.

**TABLE 5:** Number of hESC and hiPSC lines registered in some Stem Cell Banks and Registries (as of December, 2021)

Bank/ Registries	Name	Country	Website	Number of hPSC lines
B	BLCB	Spain	https://www.isciii.es/	212
R	hPSCreg	EU (Germany)	https://hpscreg.eu/total/validated	7400/3771
B/R	HipSci	United Kingdom	http://www.hipsci.org/	840
B	EBiSC	Germany	https://ebisc.org/	896
B/R	CIRM/FufiFilm	United States	https://fujifilmcdi.com/the‐cirm‐ipsc‐bank/	1554
B	WiCell Research Institute	United States	https://www.wicell.org/	1541
B/R	eagle‐i	United States	https://www.eagle‐i.net/	2415
B/R	RIKEN BRC	Japan	https://www.brc.riken.jp/en/	6178
R	SKIP	Japan	https://skip.stemcellinformatics.org/	5909
B	Taiwan Human Disease iPSC Service Consortium	Taiwan	https://catalog.bcrc.firdi.org.tw/	109
B	National Stem Cell Bank of Korea	Korea	http://kscr.nih.go.kr/	154
R	Cellosaurus	Switzerland	https://web.expasy.org/cellosaurus/	5803

*Note*: The numbers refer to hPSC lines only, although some banks listed in the table also harbour non‐hPSC lines; WiCell: Neural stem cells, embryonic carcinoma stem cell lines; RIKEN: Animal iPSC and ESC lines, germline stem cell lines, cord blood stem cells and others; SKIP: Somatic stem cells, cancer stem cell, others; Cellosaurus: All cell lines.

Several attempts have been taken to improve the data sharing in stem cell line field, including proposing utilization of standardized data items and formats.^5^, ^15^, ^16^

Many of the stem cell banks and registries have similar aims: to provide a centralized resource of data for stem cells, where the data are housed in a standardized, curated and searchable format.^14^ [Finkelstein, 2018]. In order to facilitate this goal in a wider scale, hPSCreg has been improving the FAIRness of the stem cell line data according to FAIR principles. When applied, these principles will provide an efficient framework for stem cell data sharing among different banks and registries. Currently, hPSCreg is sharing stem cell line data with other databases upon request using an APIs. Data from hPSCreg are also utilized by the integrated collection of stem cell banked data (ICSCB; https://icscb.stemcellinformatics.org/).^17^

However, even with the standard nomenclature and standard data formats, data exchange in the field of stem cell lines still faces the diverse regulatory landscapes of different countries. It would be increasingly significant for application of stem cell lines in further research, drug screening, cohort studies or clinical application to implement efficient and effective data sharing among all different stem cell banks and registries. To achieve this, efficient and workable mechanisms must be developed to comply with national regulatory requirements and at the same time allow data access and use. For example, China does not permit export of human genetic data in principle. Conditions are currently being explored for data sharing and data access provisions between hPSCreg and the Chinese National Stem Cell Recourse Center (NSCRC). Here, the distinction between permissible and sensitive data needs to be defined and handling and sharing procedures for sensitive data developed in a mutual process.

Another strategy to share sensitive data may be the development of advanced data encryption technologies. This may enable to share the information embedded in the data, but not the sensitive data themselves. hPSCreg is currently exploring pilot applications of such technologies. This feature will greatly improve the searchability of individual stem cell lines, and increase the potential of the banked cell lines being applied to genome‐related research.

To build an integrated data system, FAIR data standards must be implemented throughout the stem cell data area, including Registries, Cell Banks and publications. This must go in synch with improved search and filter capabilities. These may include searching for ethical provenance details, specific mutations or nucleotide strings, finding related lines or selected cohorts based on genetic or phenotypic features. Here, hPSCreg closely collaborates with other EU‐consortia such as the EJP‐RD and EuroGCT and their resources. Inclusive collaboration is needed to move forward not only in data collection and organization, but in their access via a data and knowledge sharing hub including navigation instructions through the various legal and regulatory landscapes. For that, the identification of gaps, needs and road blocks requires direct communication, including discussion on ethics, data formats and standards, IT systems and applicable quality control assays. The setting of priority action within the stakeholder community will be needed to maximize benefit in a coordinated programme.

## 10 FUTURE ROLE OF HPSCREG AND EMERGING DISRUPTIVE TECHNOLOGIES

Pluripotent stem cells are genetically identical to their donor and thus represent miniature biological twins of the global donor population. The more extensively these cells are donated, the more detailed they reflect the genetic diversity of the global population. Stem cells are also, in principle, biological and functional representatives of their donors. They can be differentiated into increasingly complex tissue structures such as organoids mimicking the donor's morphological, physiological and functional properties. The combination of organoids of different tissues allow the modelling of ever more complex bodies. With the possibility to generated embryo‐like entities from hiPSC and hESC, the whole organism is modelled in an early developmental state.

**FIGURE 2:**
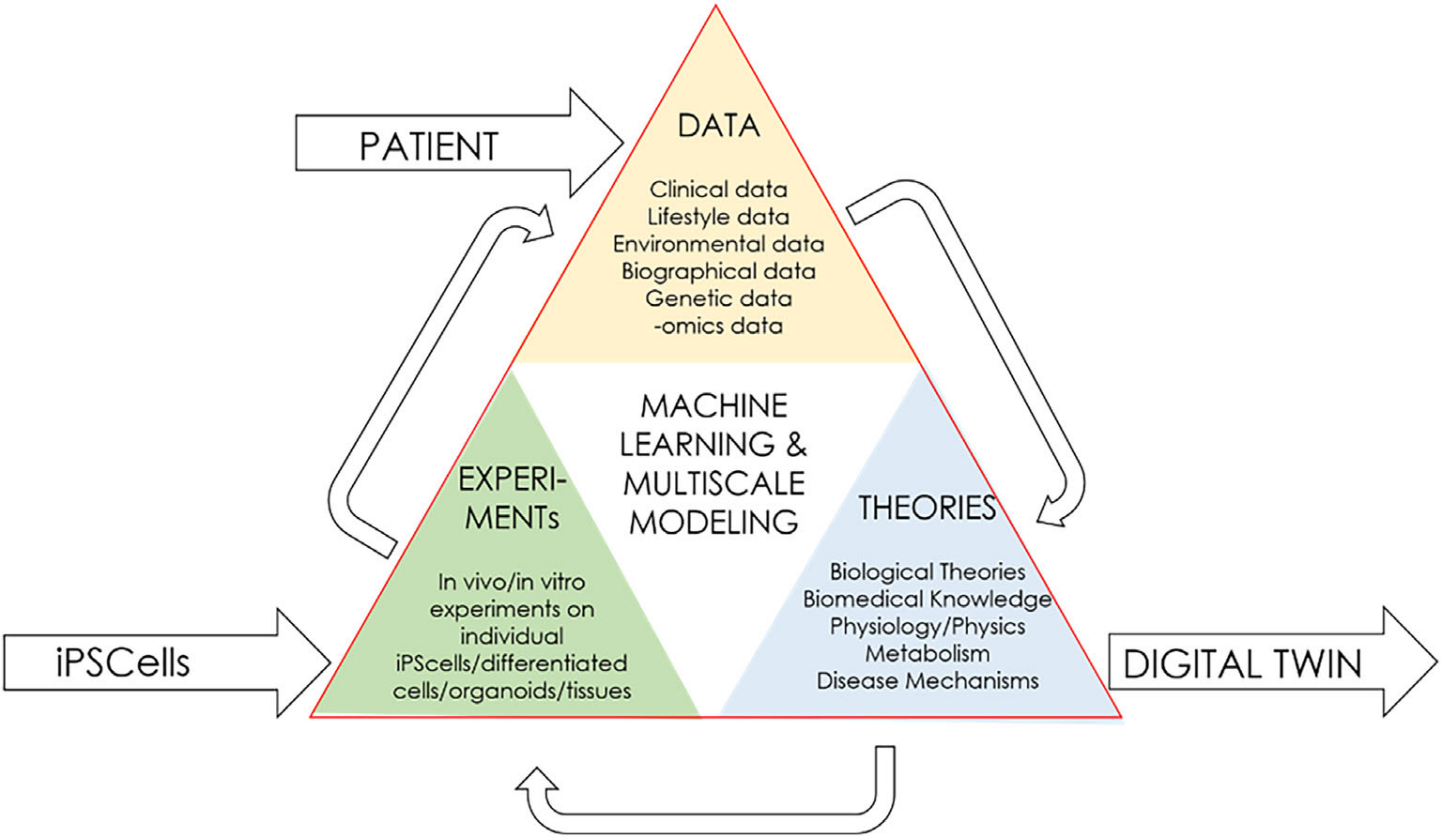
Components of a bio‐data hybrid

Since, in addition to the genetic data contained in the stem cells themselves, a variety of other data from the donors is also recorded and collected (lifestyle data, environmental and biographic data, gender, ethnicity, [rare] diseases) so other aspects of global population are also included in connection with stem cell collections. From this data, it is possible to develop a digital model of the original donor with the help of artificial intelligence (AI) systems. These digital models are called digital twins, which is the virtual simulation of cells, organoids, organs and even whole organisms developed using machine learning, multiscale modelling and other AI programmes. With their help, diverse reactions of cells, organs and organisms can be simulated and predicted through virtual representation. These digital models can, for example, reveal hidden patterns or connections and disease mechanisms.^18^ However, the value of these digital models hinges on their verifiability: using stem cells, which are miniature biological twins of donors, the digital model can be tested on the biological model without harming the patient himself. Stem cells can be turned into differentiated cells, organoids, organs that can serve as patient's biological representatives to verify digital models in vivo.^19^ Viewed as a unit, the stem cell twin of a person and the digital twin form a trainable bio‐data hybrid that can represent individual patients in ever‐increasing complexity and thus be used in personalized medicine. A donor's bio‐data hybrid hence would consist of all the data associated with the person, the general scientific knowledge of human physiology, metabolism and disease mechanisms, and his personal stem cells. These cells, in turn, help to generate further data through experimentation so that the digital models and simulations that could be developed from this data through machine learning can be improved and tested (Figure 2).

It is foreseeable that repositories of hPSC data and cell banks may take over a much more active role in mediating between the donors of the lines and the user and researcher. There is an increasing need by donors to information regarding the use of their donated cells, and data. This interest in further increased by the emergence of new applications and technologies such as blastoid and organoid research, and web‐wide AI applications. hPSCreg can play an intermediary role in this technical development by collecting, referencing and presenting data associated with cell lines and the global availability of stem cell lines, thus becoming an important information link between users and donors or patients in a larger data ecosystem. By specifically addressing ethical challenges in the field, hPSCreg aims to support donor empowerment and patient engagement, as well as implementing measure to protect donor privacy and the preservation of the informed consent process. The implementation of clear standards in this area is not only necessary to give donors the chance to influence utilization of their data and to receive feedback about achieved results. This will at the same time provide reassurance for researchers venturing into new areas of science.

## 11 HPSC‐DERIVED CELLS

Pluripotent stem cells are the source material to generate a plethora of derived more differentiated and specialized cell types for research and diverse applications, including clinical and commercial use. To implement the biological twin concept, it will be essential to provide information and data about the cells derived from specific PSC lines. Again, standards and descriptors for cell type identity, quality assessment, genetic and epigenetic characterization needs will be necessary to enable a usable connection between data from donors, iPSC and derived cells. While several projects exist to develop matrices for cell type definition,^20^, ^21^, ^22^ these need to be linked to the PSC community. hPSCreg currently develops pilots in this direction in collaboration with the EBiSC iPSC bank.

## AUTHOR CONTRIBUTIONS

AK Contributed in conception, design, interpretation of data. NM Drafting and revising of manuscript. YC Contributed with acquisition of data and drafting the manuscript. AF Drafting and revising of manuscript, interpretation of data. SK Contributed with acquisition of data and drafting the manuscript. SS Contributed with acquisition of data and drafting the manuscript. SM Contributed in conception, design, interpretation of data.

## Data Availability

Data sharing is not applicable to this article as no new data were created or analyzed in this study.

## References

[1] Borstlap J , Stacey G , Kurtz A , et al. First evaluation of the European hESCreg. Nat Biotechnol. 2008;26(8):859‐860. 18688231 10.1038/nbt0808-859

[2] Seltmann S , Lekschas F , Müller R , et al. hPSCreg–the human pluripotent stem cell registry. Nucleic Acids Res. 2016;44(D1):D757‐D763. 26400179 10.1093/nar/gkv963 PMC4702942

[3] Mah N , Seltmann S , Aran B , et al. Access to stem cell data and registration of pluripotent cell lines: the human pluripotent stem cell registry (hPSCreg). Stem Cell Res. 2020;47:101887. 32707486 10.1016/j.scr.2020.101887

[4] Wilkinson M , Dumontier M , Aalbersberg I , et al. The FAIR guiding principles for scientific data management and stewardship. Sci Data. 2016;3:160018. 26978244 10.1038/sdata.2016.18 PMC4792175

[5] Kurtz A , Seltmann S , Bairoch A , et al. A standard nomenclature for referencing and authentication of pluripotent stem cells. Stem Cell Reports. 2018;10(1):1‐6. 29320760 10.1016/j.stemcr.2017.12.002 PMC5768986

[6] Kobold S , Guhr A , Mah N , et al. A manually curated database on clinical studies involving cell products derived from human pluripotent stem cells. Stem Cell Reports. 2020;15(2):546‐555. 32679065 10.1016/j.stemcr.2020.06.014 PMC7419703

[7] Hao J , Ma A , Wang L , et al. General requirements for stem cells. Cell Prolif. 2020;53(12):e12926. 33146418 10.1111/cpr.12926 PMC7705904

[8] Hao J , Cao J , Wang L , et al. Requirements for human embryonic stem cells. Cell Prolif. 2020;53(12):e12925. 33073898 10.1111/cpr.12925 PMC7705896

[9] Isasi R , Namorado J , Mah N , Bultjer N , Kurtz A . A pathway for attesting ethical provenance of cell lines: lessons from the European human pluripotent stem cell registry (hPSC^reg^). Stem Cell Res. 2019;40:101539. doi:10.1016/j.scr.2019.101539 31450190

[10] Lomax GP , Hull SC , Isasi R . The DISCUSS project: revised points to consider for the derivation of induced pluripotent stem cell lines from previously collected research specimens. Stem Cells Transl Med. 2015;4(2):123‐129. 25561681 10.5966/sctm.2014-0192 PMC4303360

[11] Caulfield T , Ogbogu U , Nelson E , et al. Stem cell research ethics: consensus statement on emerging issues. J Obstet Gynaecol Can. 2007;29(10):843‐848. 17915069 10.1016/s1701-2163(16)32632-9

[12] Bairoch A . The cellosaurus, a cell‐line knowledge resource. J Biomol Tech. 2018;29(2):25‐38. 29805321 10.7171/jbt.18-2902-002 PMC5945021

[13] Statements on Regulation (EU) 2021/695 of the European Parliament and of the Council of 28 April 2021establishing Horizon Europe – the Framework Programme for Research and Innovation, laying down its rules for participation and dissemination, and repealing Regulations (EU) No 1290/2013 and (EU) No 1291/2013(1) (2021/C 185/01)

[14] Chen Y , Sakurai K , Maeda S , et al. Integrated collection of stem cell bank data, a data portal for standardized stem cell information. Stem Cell Reports. 2021;16:997‐1005. 33740463 10.1016/j.stemcr.2021.02.014 PMC8072026

[15] Luong MX , Auerbach J , Crook JM , et al. A call for standardized naming and reporting of human ESC and iPSC lines. Cell Stem Cell. 2011;8(4):357‐359. 21474098 10.1016/j.stem.2011.03.002

[16] Sakurai K , Kurtz A , Stacey G , Sheldon M , Fujibuchi W . First proposal of minimum information about a cellular assay for regenerative medicine. Stem Cells Transl Med. 2016;5(10):1345‐1361. 27405781 10.5966/sctm.2015-0393 PMC5031183

[17] F J , Parvanova I , Zhang F . Informatics approaches for harmonized intelligent integration of stem cell research. Stem Cells Cloning. 2020;13:1‐20. 32099411 10.2147/SCCAA.S237361 PMC6996484

[18] Barricelli BR , Casirighi E , Fogli D . A survey on digital twin: definitions, characteristics, applications, and design implications. IEEE Access. 2019;7:167653‐167671. doi:10.1109/ACCESS.2019.2953499

[19] Kim J , Koo BK , Knoblich JA . Human organoids: model systems for human biology and medicine. Nat Rev Mol Cell Biol. 2020;21(10):571‐584. doi:10.1038/s41580-020-0259-3 32636524 PMC7339799

[20] Osumi‐Sutherland D , Xu C , Keays M , et al. Cell type ontologies of the Human Cell Atlas. Nat Cell Biol. 2021;23:11‐1135. 34750578 10.1038/s41556-021-00787-7

[21] Börner K , Teichmann SA , Quardokus EM , et al. Anatomical structures, cell types and biomarkers of the Human Reference Atlas. Human Nat Cell Biol. 2021;23:1117‐1128. 10.1038/s41556-021-00788-6 PMC10079270 34750582

[22] Panina Y , Karagiannis P , Kurtz A , Stacey GN , Fujibuchi W . Human Cell Atlas and cell‐type authentication for regenerative medicine. Exp Mol Med. 2020;52(9):1443‐1451. 32929224 10.1038/s12276-020-0421-1 PMC8080834

